# The Ideal Man

**Published:** 1904-09

**Authors:** J. V. Conzett

**Affiliations:** Dubuque, Iowa.


THE AMERICAN
### JOURNAL OF DENTAL SCIENCE

### THE IDEAL MAN.

By J. V. CONZETT, D. D. S., Dubuque, Iowa.

(Written for *The American Journal of Dental Science.*)

In this practical, utilitarian age, ideals are so frequently relegated to the rear, and indeed usually never thought about, that it would take a trumpet call to cause us to lift our eyes and hearts from the sordid things of earth that we are so earnestly pursuing, to the grander though invisible, things of Heaven.

The Idealist, the Dreamer of dreams, the Poet, living as he does in fancy, above the clouds of dust and fog that obscure the vision of the ordinary man, sees into the future and dreams of conquests of nature that seem at the time but idle dreams. But in time the scientist, following in his footsteps, but may hap afar off, by the alchemy of his genius turns the dream of the Poet into the substantial discovery of the scientist. And the world applauds the one because it can use the product of his labor, while it has long since forgotten the other, who has made possible the present success. Kant in his "Critique of Pure Reason" says, "While the idea rules, the ideal serves as the architype for the permanent determination of the copy, and we have no other rule of our actions, but the conduct of the divine man within us, with which we compare ourselves, though we can never reach it. These ideals, though they can never claim objective reality, are not therefore to be considered as chimeras, but supply reason with an indispensable standard, because it requires the concept of that which is perfect of its kind, in order to estimate and measure by it the degree and number of the defects of the imperfect."

When Emerson said "Hitch your wagon to a star," he had no thought that any man would attain the stellar goal, but he knew that the man whose ideals were ever in the heavens, would continually mount upward, and the farther he went tasting the increasing joys of his altitude, the more earnestly would he press on toward his goal.

I have no apology then to make for presenting some of the ideals that have appeared unto me in vision. And if they are unattainable with the present means of locomotion, if with our infinite limitations we can not realize the absolute of our dreams, we know that the striving for them will bring us nearer our goal, and the vision conceived of our desire, with infinite labor will bring forth in the eternities the realities that present are but dreams and desires.

The Ideal Dentist must first be an Ideal Man. We can all agree upon the proposition that man has a triune nature, Physical, Intellectual and Spiritual, for even those that are sceptical concerning the existence of the soul must accept the dictum of the Psychologist, that the mind has a dual nature, viz., objective and subjective; and the subjective mind has all the attributes of the soul. The Ideal Man then must have a perfectly developed body, mind and soul.

It is scarcely necessary to emphasize the need of physical development, for with the gymnasiums and physical directors scattered over the land, and the magazines filled with advertisements of devices that will enable every man to be his own trainer, we are in danger of going to the extreme of over, rather than under-development. Still as Dentists we are apt to overlook the needs of the body. Every man should strive to have a perfect body, for without a good physical development, the highest mental effort is impossible. Alexander Stevens was a man of gigantic intellect, hampered by an invalid body, and Herbert Spencer, magnificent intellectual giant that he was, could work only two hours a day, because of physical weakness. What might those men have accomplished, had they possessed perfect bodies?

To the Dentist who must do the most arduous labor from eight to ten hours a day, a strong physique is an absolute necessity. I might cite instance after instance of failures that have occurred, simply because the body was not able to bear the burden that the duties of the profession demanded. The Ideal Man will be strong physically because he will care for his body, not only by exercising its various muscles properly and observing the laws of development and hygiene, but by abstaining from those things that will have a detrimental effect upon its functions and habits. I have no desire at this time to preach a temperance sermon, although from the evidence gathered at some of our conventions, together with the wrecks I have observed at various times, it would not be out of place. But the Ideal Dentist will not use alcoholic beverages or tobacco; first, because stimulants and narcotics are unnatural, and detract from that perfect poise of nerve and muscle that is so essential to operative success, and second, because the odor of alcoholic drinks and tobacco is extremely distasteful to the lady or gentleman of refinement.

The laws that govern the development of the body apply with equal force to the development of the intellect. In order to have physical growth, we must have food and exercise. The food must be of proper kind and amount and must be digested and properly assimilated, and the exercise must be such that all muscles shall have their proportionate amount. "Unto him that hath shall be given, but unto him that hath not shall be taken away even that which he hath," is applicable to all phases of individual development. If a muscle, organ, nerve or brain cell is neglected and not used it will atrophy and become useless. So with the development of the intellect; it must have food of the proper kind and amount, which must be properly digested and assimilated; and it must have proper exercise.

The Ideal Man will find his intellectual pabulum in study and investigation. He will read, for "reading maketh a full man," says Bacon, and will digest well that which he reads, assimilating the good and true and eliminating the false and untrue. He will exercise his intellectual faculties in investigating the secrets of nature, and "read God's thoughts after Him." He will appreciate the full force of the saying of the writer of Proverbs, "as he thinketh in his heart, so is he," and will be very careful to think right thoughts.

In this day of sensuality and of so much of moral uncleanness it behooves the dentist especially to guard well the secret chambers of his thought, for that which is thought in secret will be done in the open. A man is what he desires to be, and will eventually so show himself to the world. Luther said, "We cannot help the birds flying above our heads, but we can prevent their making their nests in our hair." So we cannot help evil thoughts and foul suggestions coming to us, but we can prevent their abiding with us. Be clean in thought and you will be clean in action. Be unclean in thought and the end shall find you a moral leper.

"Sow a thought and you reap a deed,
Sow a deed and you reap a habit,
Sow a habit and you reap a character,
Sow a character and you reap a destiny."

Man's destiny then is that which he thinks. The Ideal Man will have no place in his thoughts for anything that is foul or unclean or for anything that "maketh a lie." He will have a symmetrically developed mind for he will exercise all portions of his mind. He will "hold high converse with the mighty dead" by reading the literature of past ages, singing the songs of Zion with David, and of ancient Heles with the Blind Homer, as well as reveling in the writings of the modern masters of song. He will be conversant with the history and the art of the world, while the masters of science will be his familiar friends.

With the duties of one's profession and with the technical reading that we must do, the question frequently asked is when shall we find the time for general reading. The answer is that the wasted minutes if properly used, will more than suffice to give any man a liberal acquaintance with literature in general. Have always at hand some book that you can pick up when you are resting, and utilize the time that would otherwise be wasted, in storing up in the chambers of memory some gem of thought that afterwards will be of priceless value to you. In this way there will be attained in a few years a knowledge of history, biology, psychology, music, etc., etc., that will be a surprise and pleasure to you. "Keeping everlastingly at it brings success," and I know of no other way to accomplish it.

Who was it said that "genius is simply a capacity for hard work?" In that sentence he struck out a truth that it would be well for us all to take to heart. Henry Drummond has said that natural laws can be projected into the spiritual world, and that spiritual life is amenable to the same laws that govern natural life. But first we must have the life. Dr. Hudson, in his book on "The Laws of Psychic Phenomena," says that when Jesus declared, "He that believeth * * * hath everlasting life," he stated a truth that science has demonstrated after these nineteen hundred years. The belief of the objective or reasoning mind in the immorality of the soul and the audible expression of that belief, give to the subjective mind or soul the suggestion that calls it into conscious life. This is in accord with the text, "With the heart man believeth unto righteousness and with the mouth confession is made unto salvation." Romans, 10 : 10.

There are many who have no conception of spiritual life, and are like the man who exclaimed when he heard two Christians in conversation, "You fellows are crazy; I don't understand what you are talking about," and one turned to him and said, "No, you cannot, for these things are spiritually concerned." The blind cannot appreciate a masterpiece of art, nor the deaf an oratorio, and yet it would be folly for the blind to say, "Because I cannot see it, it does not exist," or for the deaf to say, "There is no such thing as harmony." But that is just the logic of the man, who because he cannot discern it, says, "There is no God and no future life." The Ideal Man will as carefully, yea more carefully, culture his spiritual nature, than his intellectual or his physical nature, for he will appreciate the fact that the spirit is above mind and body, and that the body and mind have simply been called into existence for the culture of that part of man which shall endure when the "Heavens and earth shall pass away." The spirit is fed and nurtured by communion, meditation, and prayer, with and to the Great Universal Spirit,—Herbert Spencer's "Great Unknown," the Christian's God; and is developed and made strong by exercising its functions in eliminating evil from the life and in serving its fellow man, which, some one has said, is the best service of God.

The truly spiritual man is the unselfish man, the man who is always striving for the good of others, the man who has learned and practices the Golden Rule, "Whatsoever ye would that men should do to you, do ye even so to them."

In a paper of this description one can but touch upon the subject, leaving the amplification thereof to the reader; and with the hope that some one may be led to think on these things and from thinking be led to desire a larger share of the Ideal Life, I submit in all humility these thoughts on that which is so far above me that I hesitate to send them forth.

